# The Apoptotic Function Analysis of p53, Apaf1, Caspase3 and Caspase7 during the Spermatogenesis of the Chinese Fire-Bellied Newt *Cynops orientalis*

**DOI:** 10.1371/journal.pone.0039920

**Published:** 2012-06-29

**Authors:** Da-Hui Wang, Jian-Rao Hu, Li-Ya Wang, Yan-Jun Hu, Fu-Qing Tan, Hong Zhou, Jian-Zhong Shao, Wan-Xi Yang

**Affiliations:** 1 The Sperm Laboratory, College of Life Sciences, Zhejiang University, Hangzhou, People's Republic of China; 2 College of Life and Environmental Sciences, Hangzhou Normal University, Hangzhou, People's Republic of China; 3 Department of Reproductive Endocrinology, The Women’s Hospital, School of Medicine, Zhejiang University, Hangzhou, People's Republic of China; 4 The First Affiliated Hospital, College of Medicine, Zhejiang University, Hangzhou, People's Republic of China; St. Georges University of London, United Kingdom of America

## Abstract

**Background:**

Spontaneous and stress-induced germ cell apoptosis during spermatogenesis of multicellular organisms have been investigated broadly in mammals. Spermatogenetic process in urodele amphibians was essentially like that in mammals in spite of morphological differences; however, the mechanism of germ cell apoptosis in urodele amphibians remains unknown. The Chinese fire-belly newt, *Cynops orientalis*, was an excellent organism for studying germ cell apoptosis due to its sensitiveness to temperature, strong endurance of starvation, and sensitive skin to heavy metal exposure.

**Methodology/Principal Findings:**

TUNEL result showed that spontaneous germ cell apoptosis took place in normal newt, and severe stress-induced apoptosis occurred to spermatids and sperm in response to heat shock (40°C 2 h), cold exposure(4°C 12 h), cadmium exposure(Cd 36 h), and starvation stress. Quantitative reverse transcription polymerase chain reactions (qRT-PCR) showed that gene expression of *Caspase3* or *Caspase7* was obviously elevated after stress treatment. *Apaf1* was not altered at its gene expression level, and *p53* was significantly decreased after various stress treatment. Caspase assay demonstrated that Caspase-3, -8,-9 enzyme activities in newt testis were significantly elevated after heat shock (40°C 2 h), cold exposure(4°C 12 h), and cadmium exposure(Cd 36 h), while Caspase3 and Caspase8 activities were increased with Caspase9 significantly decreased after starvation treatment.

**Conclusions/Significance:**

Severe germ cell apoptosis triggered by heat shock, cold exposure, and cadmium exposure was Caspase3 dependent, which probably involved both extrinsic and intrinsic pathways. Apaf1 may be involved in this process without elevating its gene expression. But starvation-induced germ cell apoptosis was likely mainly through extrinsic pathway. p53 was probably not responsible for stress-induced germ cell apoptosis in newt testis. The intriguing high occurrence of spermatid and sperm apoptosis probably resulted from the sperm morphology and unique reproduction policy of Chinese fire-belly newt, *Cynops orientalis.*

## Introduction

Apoptosis is evolutionarily conserved, genetically driven and energy-dependent programmed cell death, which plays pivotal roles in development and homeostasis of multicellular organisms [1], [2]. A large body of evidence proved the great importance of apoptosis for spermatogenesis in vertebrates and invertebrates [3], [4], [5], [6], [7], [8]. Spermatogenesis is a well-organized complex process, in which diploid spermatogonia proliferate and differentiate into terminally haploid mature functional sperm. However, during normal spermatogenesis not all spermatogonia in the testis can undergo spermiogenesis and become mature sperm, for most of them are eliminated through spontaneous germ cell apoptosis [7], [8], [9]. Notably, germ cell apoptosis can be induced by various environmental or physiological stress, such as heat shock, UV irradiation, toxicant exposure, hormonal withdrawal and oxidative stress [3], [10], [11], [12], [13], [14], [15], [16]. Both spontaneous apoptosis and stress-induced apoptosis are indispensible for healthy germ cell development and functional sperm maturation [17]. Above all, apoptosis maintains cellular homeostasis and a suitable ratio of Sertoli cells to germ cell. Furthermore, apoptosis serves to eliminate potentially defective germ cells and ensure high-quality gametes production [18], [19], [20]. Finally, apoptosis keeps germline genetic integrity by clearing off mutant cells and prevents transmission of mutations to progeny and offspring [21], [22], [23].

Two different mechanisms have been elucidated for cell apoptosis: the extrinsic receptor-mediated pathway and the intrinsic mitochondria-dependent pathway. The extrinsic pathway was initiated with the extracellular death ligands, like Fas ligand (FasL), binding to their corresponding cell-surface receptors such as Fas. Then a death-inducible signaling complex (DISC) formed, leading to the activation of caspase8. Eventually, the activated caspase8 triggered activation of downstream effector caspases: caspase3, 6 and 7 that were directly responsible for cell demise [24], [25], [26]. By contrast, the intrinsic pathway was mainly regulated by Bcl-2 family members including Bax, Bak, Bcl-2, Bcl-xl and so on [21], which could positively or negatively regulate mitochondrial outer membrane permeabilization to promote the release of cytC (cytochrome C) and other apoptotic molecules. In cytosol, cytC, Apaf1 and procaspase9 were assembled into apoptosome to activate downstream effector caspase3, 6 and 7, leading to cellular components degradation and cell death [25]. Both the extrinsic and intrinsic pathways proved to participate in germ cell apoptosis during spermatogenesis. On the one hand, Bax-mediated apoptosis was indispensable for spermatogenesis [21]. Bax was elucidated to be essential for the progression of the first wave of spermatogenesis in mice [27], and it was found highly expressed in testis of young mice but only expressed at low level in spermatogonia of adult mice [28], [29]. The Bax-null male mice were infertile as a result of disordered germ cell apoptosis, which caused excessive numbers of spermatogonia and pre-leptotene spermatocytes in spermatogenesis [13], [21], [30]. On the other hand, Fas and FasL were also evidenced essential for germ cell apoptosis during spermatogenesis. Fas was expressed in germ cells and FasL detected in Sertoli cells [31], [32], [33], [34]. Testicular Fas participated in germ cell apoptosis triggered by testosterone withdrawal [35], UV radiation [36] and toxicants exposure [32], [36].

Both the two distinct caspase-mediated apoptosis pathways could be regulated by p53. p53 was the most prominent tumor suppressor, and many types of cancers resulted from functionally impaired p53 [37], [38] that lost its capability in DNA repairing and cell apoptosis induction. It was demonstrated that p53 played critical functions in germ cell apoptosis both through extrinsic pathway and intrinsic pathway, since it could regulate not only Bax, Bid, Bcl-2, Bcl-xL, Puma, Noxa, Apaf1 and IAP, but also death receptors like CD95, Fas, Apo-1 and DR5 [39], [40], [41], [42], [43], [44]. p53-mediated apoptosis was found responsible for the initial phase of germ cell apoptosis during spermatogenesis in cryptorchid mice [31], [45], [46]. Moreover, under genotoxic stress, p53 was involved in apoptosis of spermatogonia, spermatocytes and spermatids [39], [40], [41]. A higher percentage of morphologically abnormal spermatozoa in the ejaculate and reduced fertility occurred to *p53*−/− mice, which was probably due to failed germ cell apoptosis [45].

Previous researches on germ cell apoptosis mainly focused on mammals for an elaborate and comprehensive knowledge of mammalian spermatogenesis. Actually, the spermatogenetic process in urodele amphibians is essentially like that in mammals in spite of morphological differences. During the spermatogenesis of *Cynops orientalis*, spermatogonia underwent several cycles of cell division to become spermatid, and later in spermiogenesis, the spermatid eventually matured into sperms. Mature sperm commonly contained typical acrosome, long and narrow nucleus and long flagellum, and a large number of mitochondria were arranged around the midpiece of the sperm flagellum. Like other newt species, the testis of *Cynops orientalis* consists of lobules in successive zones, and spermatogenesis proceeds synchronously in these zones. Therefore, it is easy to isolate and identify germ cells of particular spermatogenic stage, which is very suitable for germ cell apoptosis analysis (unpublished data). As an important urodele amphibian, the Chinese fire-belly newts, *Cynops orientalis*, is an excellent organism for studying germ cell apoptosis due to its advantageous physiological features: sensitiveness to temperature, strong endurance of starvation and sensitive skin to environmental heavy metal. Coincidentally, temperature, starvation and heavy metals are often utilized to investigate germ cell apoptosis. Besides, germ cell apoptosis have been reported in Japanese fire-belly newts and marbled newts, and some unidentified caspase was reported involved in germ cell apoptosis in Japanese fire-belly newts [47], [48], [49], [50], [51], [52], [53], [54]. However, the detailed mechanism of germ cell apoptosis was not elucidated. In the present study, we investigated the germ cell apoptosis induced by heavy metal exposure, starvation and temperature stress in Chinese fire-belly newts, *Cynops orientalis*. We provided evidence that stress-induced germ cell apoptosis occurred by activating Caspase3 both through extrinsic pathway and intrinsic pathway. In addition, Apaf1 probably was involved in the stress-induced germ cell apoptosis, whereas p53 was not likely responsible for this process, which needs further investigation.

## Materials and Methods

### Animals and Sampling

This study didn’t involve non-human primates. Research work was performed in full accordance to the requirement by ‘Governing Regulation for the Use of Experimental Animals in Zhejiang Province’ (Zhejiang Provincial Government Order No 263, released in August 27, 2009, effective from October 1, 2010) and ‘Governing Regulation for the Use of Experimental Animals in Zhejiang University’ (Zhejiang University Order No 1 (2009), released in November 24, 2009). Fire-belly newts used in this study were purchased from local pet market of Fuyang Moutainous Area. The use of this animal was approved by local review boards for ethics in College of Life Sciences, Zhejiang University.

Adult Chinese fire-belly newts, *Cynops orientalis,* were collected from Fuyang Mountainous Area (Hangzhou, China). Animals were kept in big aquatic tank in laboratory (12 h light:12 h darkness at 22±2°C. All the animals were anesthetized by placing them for 20 min in a solution of 0.1% MS222 (Sigma, St. Louis). Fresh samples of testis, liver, muscle, intestine and heart were dissected and stored at -80°C for total RNA extraction.

### Full-length cDNA Cloning of *p53*, *Apaf1*, *Caspase3* and *Caspase7*

Total RNA from the testis of adult Chinese fire-belly newt, *Cynops orientalis*, was extracted using Trizol (Invitrogen, USA) according to the instruction book, and reverse transcription was performed using BioRT cDNA First Strand Synthesis Kit (Bioer, Japan). Degenerate primers were designed using CODEHOP program supplied by Blocks WWW Server (http://blocks.fhcrc.org/) based on conserved protein sequence, and gene specific primers designed by Primer 5.0 and Oligo 6. All degenerate primers and gene specific primers used for cloning *p53*, *Apaf1*, *Caspase3* and *Caspase7* were presented in **Table 1** and **Table 2**. Polymerase chain reactions (PCR) were run in Mygene Series Peltier Thermal Cyclers (Hangzhou, China), and the PCR program for amplifying cDNA fragment was as follows: 94°C for 5 min, 15 cycles in a touch down program (94°C for 30 s, 55°C for 30 s and 72°C for 30 s, followed by a 0.5°C decrease of the annealing temperature per cycle), and then 32 cycles were performed (30 s at 94°C, 30 s at 48°C and 30 s at 72°C) with 10 min at 72°C for the final extension. The RACE (Rapid amplification of cDNA ends) PCR were run according to Smart RACE cDNA Amplification Kit (ClonTech) and 3′Full RACE Amplification Kit (Takara). The target PCR products were cloned into pMD18-T vector (TaKaRa) and sequenced by Sangon Company (Shanghai, China).

**Table 1:** Primers used for the study of Caspase3 and Caspase7 in *Cynops orientalis*.

Primer name	Primer Sequence (5′to 3′)	Usage
**Csp AF1**	ATTGTATTATTATTAATaayaaraaytt	Caspase7/3 cloning
**Csp BF2**	AAGAAATGGAacngaygtnga	Caspase7/3 cloning
**Csp DR2**	GAATAAGCATACAGAAAATCNGCYTCNAC	Caspase7/3 cloning
**Csp CR1**	CCTGAATGAAAAACAGTTTNGGYTTNCC	Caspase7/3 cloning
**Csp CF1**	ACCAAAACTGTTCTTTATTcargcntgymg	Caspase3 cloning
**Csp3lF1**	AAAGTTTTCAATGACCAAGC	Caspase3 cloning
**Csp3lR1**	ATCTGACGAATCTCCTCCAC	Caspase3 cloning
**Csp7-3′gspF1**	CTTATCTATGGGACAGACGGGC	Caspase7-3′RACE
**Csp7-3′gspF2**	GGTGAAGAGGGACTTATCTATGGG	Caspase7-3′ RACE
**Csp7-5′gspR1**	GCTGCTCCATTTCTTCACAGGTCCGA	Caspase7-5′ RACE
**Csp7-5′gspR2**	AGCAGGCATAATCACTGTGGTCTTCTT	Caspase7-5′ RACE
**Csp3-3′gspF1**	ATCAACAGAGGATACCCGTGGA	Caspase3-3′ RACE
**Csp3-3′gspF2**	GCAGACAGTGGAGGAGATTCGT	Caspase3-3′ RACE
**Csp3-5′gspR1**	GTAAGAAAACACTTCCTGAGATTTGCGG	Caspase3-5′ RACE
**Csp3-5′gspR2**	CCCAAGGTAAGAAAACACTTCCTGAGAT	Caspase3-5′ RACE
**Csp7rlF1**	TGGATGTTTATGTCACCTTTTC	Realtime PCR
**Csp7rlR1**	AGTCTATTTTATTATGGGCTCG	Realtime PCR
**Csp3rlF1**	AGAGAACAATGGCGGATA	Realtime PCR
**Csp3rlR1**	CCAGTTGAGGGATGAAAG	Realtime PCR
**R(A,G) Y(C,T) M(A,C) N(A,T,G,C)**		

**Table 2:** Primers used for the study of p53 and Apaf1 in *Cynops orientalis*.

Primersname	Primer Sequence(5′to 3′)	Usage
**P2F1**	TGAGAAGATGTCCACAYCAYGARMG	p53 Cloning
**P5R1**	CAAGCACACACTCKNACYTCRAA	p53 Cloning
**P5R2**	ATCTCTTCCTGGACANGCRCANAC	p53 Cloning
**P5**′**-gspR3**	CCTGCGGAGTCTCGTATGGCACCAACAC	p53 5′ RACE
**P5**′**-gspR2**	TGGCACCAACACGCTGTGGCGACGGG	p53 5′ RACE
**P3**′**-gspF3**	GCCACAGCGTGTTGGTGCCATACGAGAC	p53 3′ RACE
**P3**′**-gspF2**	CGCCGACCAATCCTGACTATCATTACAC	p53 3′ RACE
**P53RTF1**	CCCCCAAGAATGGCAAAAA	RT-PCR
**P53RTR1**	CCTGGCTCACCCCCACCTA	RT-PCR
**p53rlF1**	GATGACTGTCTCCACCCCT	Realtime PCR
**p53rlR1**	TCTGCCTTGTAATTCCCTT	Realtime PCR
**β-actinF**	GAATCCCAAAGCCAACC	Realtime PCR/RT-PCR
**β-actinR**	CCAGCCAAGTCAAGACG	Realtime PCR/RT-PCR
**Apaf2F**	TTATGAAGCTCTGGAYGARGCNAT	Apaf1 cloning
**Apaf4R2**	TTTAAAAATCTGCAGTGTTTTRTCNGCNCC	Apaf1 cloning
**Ap3**′**-gspF1**	ATGCTTGCTTTTCCCCTGATGG	Apaf1 3′ RACE
**Ap3**′**-gspF2**	GTCCGCATACAGATGGAGTTTA	Apaf1 3′ RACE
**Ap5**′**-gspR1**	GCATTTCTTCCACCTCTTCTGTCTCCA	Apaf1 5′ RACE
**Ap5**′**-gspR2**	GTGAGAAAATCCAGTTGAAGGTCGTGT	Apaf1 5′ RACE
**AprlF1**	ATGGAGTTTATCATGCTTGC	Realtime-PCR/RT-PCR
**AprlR1**	CATAGGATTAGTTCCGTTGG	Realtime-PCR/RT-PCR
**R(A,G) Y(C,T) M(A,C) K(G,T) N(A,T,G,C)**		

### Protein Alignment and Structure Prediction of p53, Apaf1, Caspase3 and Caspase7

The protein alignment and structure prediction were performed using Vector NTI10 (Invitrogen, USA) and online softwares (http://www.expasy.org/tools/scanprosite/) (http://smart.embl -heidelberg.de/)(http://zhanglab.ccmb.med.umich.edu/I-TASSER/) [55], [56], [57]. The GenBank accession numbers of Apaf1 proteins in protein alignment are as follows: *Cynops orientalis* (JQ320086); *Homo sapiens* (O14727.2); *Mus musculus* (NP_033814.2); *Xenopus laevis* (NP_001085834.1); *Danio rerio* (NP_571683.1). The GenBank accession numbers of p53 proteins used are as follows: *Cynops orientalis* (HM627863); *Homo sapiens*(BAC16799.1); *Mus musculus* (BAA82343.1); *Xenopus laevis*(CAA54672.1); *Danio rerio* (NP_571402.1).The GenBank accession numbers of Caspase7 proteins used are as follows: *Cynops orientalis* (JQ320087); *Homo sapiens* (AAC50346.1); *Mus musculus*(BAA19730.1); *Xenopus laevis* (NP_001091272.1); *Danio rerio* (AAH95327.1). The GenBank accession numbers of Caspase3 proteins used are as follows: *Cynops orientalis* (JQ320088); *Danio rerio* (CAX14649.1); *Xenopus laevis* (NP_001081225.1); *Homo sapiens* (P42574); *Mus musculus* (P70677).

### Analysis of tissue distribution of p53, Apaf1, Caspase3 and Caspase7 mRNA

Total RNA was extracted from muscle, heart, testis, intestine and liver using Trizol (Invitrogen, USA), and reverse transcription was performed using BioRT cDNA First Strand Synthesis Kit (Bioer, Japan). RT-PCR was used to analyze the gene expression level of *p53*, *Apaf1*, *Caspase3* and *Caspase7* in various tissues. The primers used for gene expression analysis were listed in Table 1 and Table 2, and the program was run as follows: 94°C for 5 min; 30 s at 94°C, 30 s at 60°C, and 30 s at 72°C (32 cycles); 10 min at 72°C for final extension. The *β-actin* served as the control, and the density of amplified bands were analyzed using Tanon GIS system (Tanon 2500).The gene expression levels of *p53*, *Apaf1*, *Caspase7* and *Caspase3* were presented as the ratio of their relative amount to *β-actin* transcripts.

### Stress challenging the Testis of Chinese Fire-belly Newt, *Cynops orientalis*

Chinese fire-belly newts were initially raised in their best conditions and then divided into several groups for stress treatments that included cadmium exposure, heat shock, cold exposure and starvation. For cadmium exposure, newts were injected subcutaneously with cadmium chloride (CdCl_2_) dissolved in saline (0.65% NaCl) according to the dosage of 50 mg/kg body weight. The newts in control group were injected only with 0.65% NaCl. Then these newts were raised for 12 h, 24 h and 36 h respectively at room temperature (22±2°C). The newts for cold exposure were kept in the incubator at 4°C (4 h and 12 h) while the newts for heat shock were exposed to 38°C (2 h, 4 h and 12 h) and 40°C (2 h and 4 h). The newts raised at room temperature served as the control. For starvation treatment, the newts were starved for one month, and those with daily feed served as the control. In each stress group, all newts were recovered in normal conditions for 4 h before their testes dissection for RNA extraction and TUNEL analysis.

**Figure 1:**
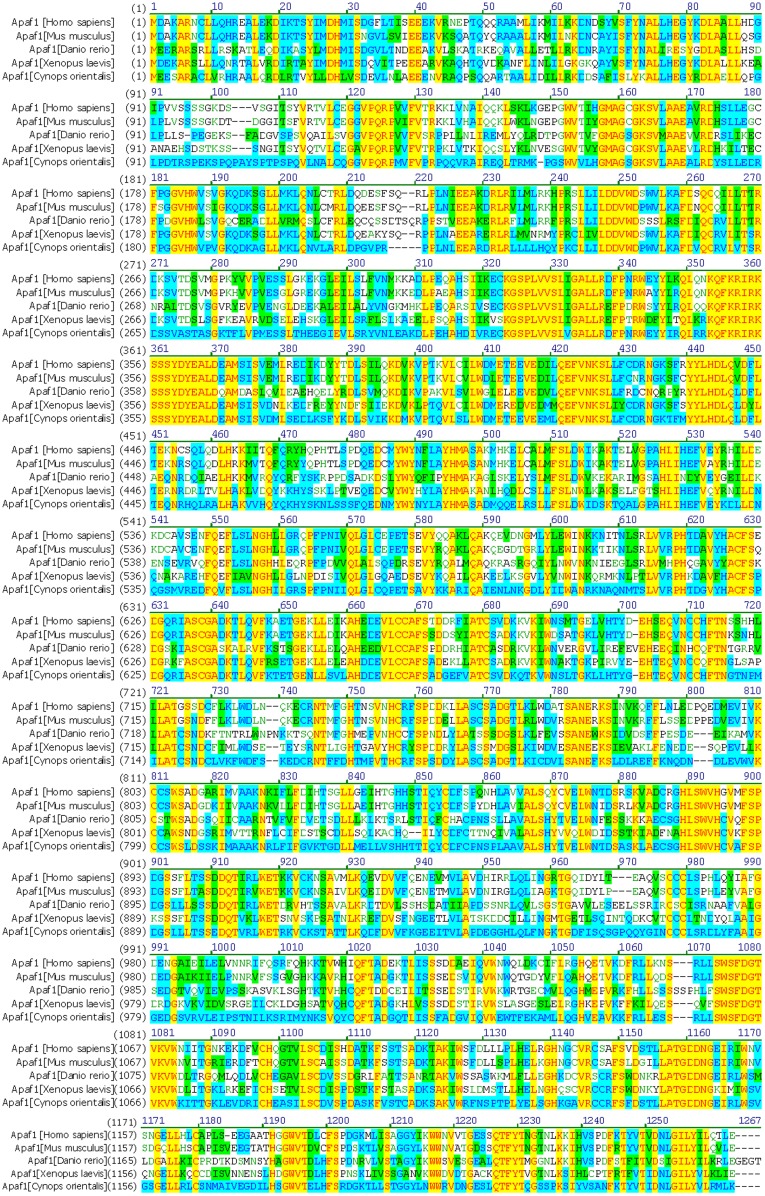
Multiple alignment of Apaf1 in *Cynops orientalis* with its homolog from several vertebrates. The protein alignment was created using Vector NTI10 (Invitrogen) with the following sequences: Cynops orientalis(JQ320086); Homo sapiens (O14727.2); Mus musculus(NP_033814.2); Xenopus laevis;(NP_001085834.1); Danio rerio(NP_571683.1). The identities of Apaf1 in Cynops orientalis with its homolog were 61.4% (Homo sapiens), 55.7% (Xenopus laevis), 51.2% (Danio rerio) and 59.9% (Mus musculus) respectively.

**Figure 2:**
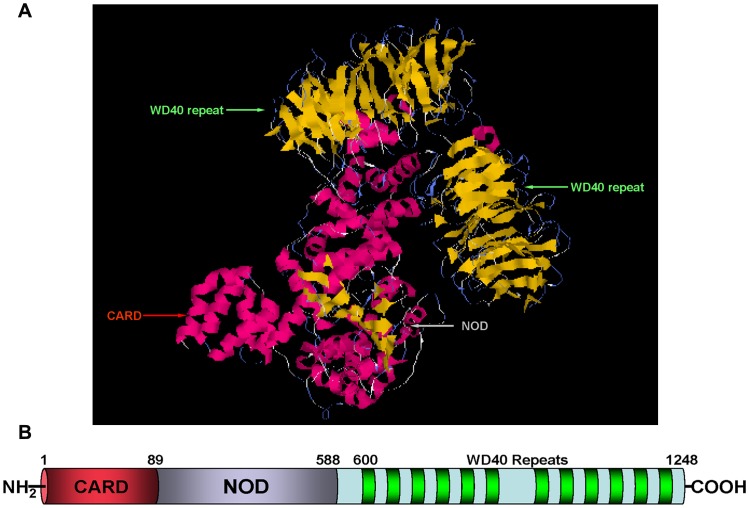
Predicted protein structure of Apaf1 in *Cynops orientalis*. (A) Cartoon representation of Apaf1 3-D structure. Yellow color stands for β-sheet and red color indicates the α-helix. The functional domains in the 3-D structures are indicated with arrows. The 3-D structure prediction was performed by I-TASSER server. (B) Schematic representation of Apaf1 subdomain structure. The subdomains are shown by the horizontal bar, with the numbers corresponding to the amino acid residues. The putative Apaf1 protein consists of three functional sudomains: the N-terminal caspase recruitment domain (CARD); the nucleotide-binding and oligomerization domain (NOD) and the C-terminal WD40 domain.

### Analysis of Gene Expression of p53, Apaf1, Caspase3 and Caspase7 after Stress Treatment

Total RNA was extracted from the testes of stress-treated newts and control newts with Trizol (Invitrogen, USA). Reverse transcription was conducted with Prime ScriptTM RT reagent kit (TakaRa, Japan) according to the instruction book. SYBR Green PCR mastermix (Takara, Japan) was used for quantitative RT-PCR, and 10 µl reaction mixture was added into Axygen 384-well reaction plates sealed with optical adhesive covers (Axygen). The cDNA levels for *p53*, *Apaf1*, *Caspase3* and *Caspase7* were normalized to *β-actin*. The primers used for realtime PCR analysis were listed in Table 1 and Table 2. The ABI PRISM 7900HT instrument (Applied Biosystems, USA) was applied for quantitative RT-PCR with each sample prepared in triplicates. The program for quantitative PCR was run as follows: 95°C for 30 s, then 40 cycles were performed (5 s at 95°C and 31 s at 60°C), and then 95°C for 15 s, 60°C for 1 min, 95°C for 15 s.

**Figure 3:**
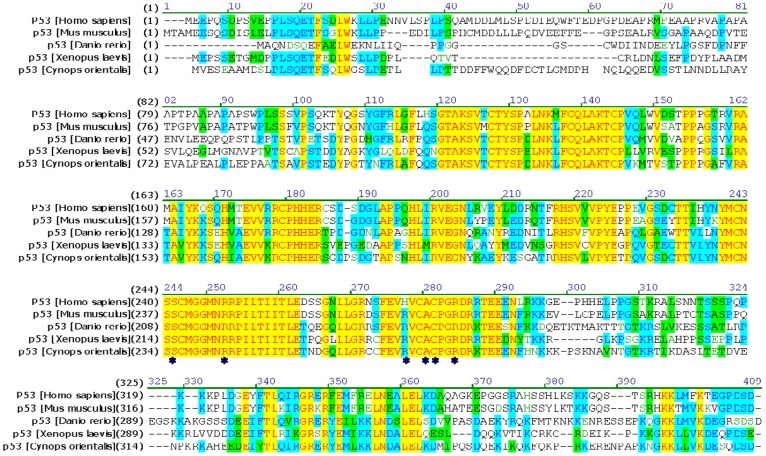
Multiple alignment of p53 in *Cynops orientalis* with its homolog from several vertebrates. The protein alignment was performed using Vector NTI10 (Invitrogen) with the following sequences: *Cynops orientalis* (HM627863)*Homo sapiens* (BAC16799.1); *Mus musculus*(BAA82343.1); *Xenopus laevis*(CAA54672.1); *Danio rerio*(NP_571402.1). The identities of p53 in *Cynops orientalis* with its homolog *were* 53.4% (*Danio rerio)*, 49.6% (*Homo sapiens)*, 48.5% (*Mus musculus)* and 52.8% (*Xenopus laevis) respectively.* Several amino acids that are essential for DNA binding are labeled with asterisks.

**Figure 4:**
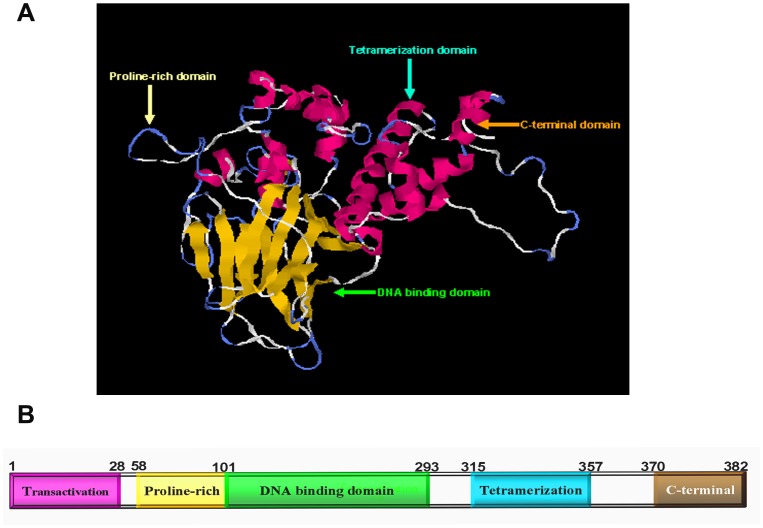
Predicted protein structure of p53 in *Cynops orientalis*. (A) Cartoon representation of p53 3-D structure. Yellow color stands for β-sheet and red color indicates the α-helix. The functional domains in the 3-D structure are indicated with arrows. The 3-D structure prediction was performed by I-TASSER server. (B) Schematic representation of p53 subdomain structure. The subdomains are shown by the horizontal bar, with the numbers corresponding to the amino acid residue. Several functional domains are in the p53: the transactivation domain, proline-rich domain, DNA binding domain, tetramerization domain and the C-terminal domain.

### TUNEL Assay

The testes of stress-treated newts and normal newts were dissected, and then wrapped in OCT compound (SAKURA, Japan) for section preparations with Micron HM525. TUNEL staining was performed on tissue sections using *in situ* cell death detection kit according to the instruction book (Beyotime, China). Briefly, the testis sections were firstly fixed using 4% paraformaldehyde (PFA) for one hour, and later washed three times with PBS. Then the sections were submerged in PBS with 0.1% Triton-X-100 in ice bath for 2 min. After further washes with PBS, the reaction was performed in the terminal deoxynucleotidyl transferase (TdT) buffer with fluorescein labeled dUTP, and the sample were incubated with reagent for one hour at 37°C with plastic membrane mounted to avoid reagent evaporation. Washed with PBS, the sections were incubated with mixture of DAPI and antifade reagent before coverglass mounting. The positive control was prepared by Positive TUNEL preparation kit (Beyotime, China), in which the sections were treated with DNase I for 30 min at 25°C and PBS wash before TUNEL labeling reaction. The TUNEL results were analyzed with a Zeiss laser scanning confocal microscope (LSM-710) (Carl Zeiss, USA).

**Figure 5:**
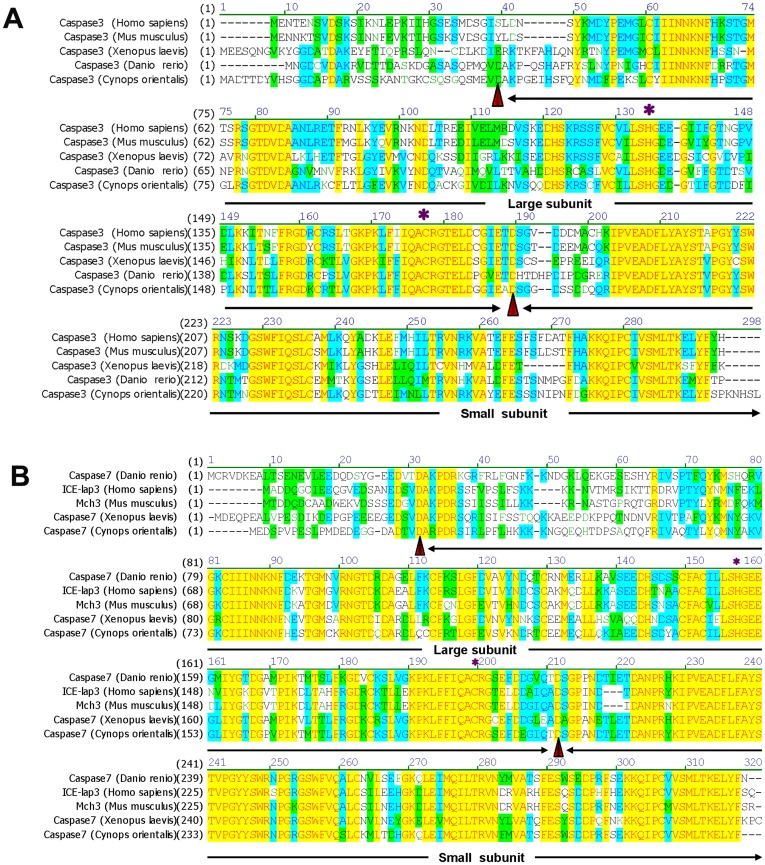
Multiple alignments of Caspase3 and Caspase7 in *Cynops orientalis* with their homolog from several vertebrates. The protein alignment was performed using Vector NTI10 (Invitrogen) with the following sequences: (A) *Cynops orientalis*(JQ320088); *Danio rerio*(CAX14649.1); *Xenopus laevis* (NP_001081225.1); *Homo sapiens* (P42574); *Mus musculus* (P70677); (B) *Cynops orientalis* (JQ320087); *Homo sapiens* (AAC50346.1); *Mus musculus*(BAA19730.1); *Xenopus laevis* (NP_001091272.1); *Danio rerio* (AAH95327.1). The identities of Caspase3 in *Cynops orientalis* with its homolog were 56.2% (*Danio rerio*), 54.4% (Xenopus laevis), 57.6%(*Homo sapiens)* and 56.3% (*Mus musculus).* The identities of Caspase7 in Cynops orientalis with its homolog were 67.6% (*Danio rerio*), 64.6% (*Xenopus laevis*), 62.6% (*Homo sapiens)* and 62.3% (*Mus musculus).* Putative cleavage sites at aspartic acid residues, which separate Caspase3 and Caspase7 into large subunits and small subunits, are labeled red arrowheads. Histines and Cystines that are essential for the catalytic centre of Caspase3 and Caspase7 are indicated by asterisk.

**Figure 6:**
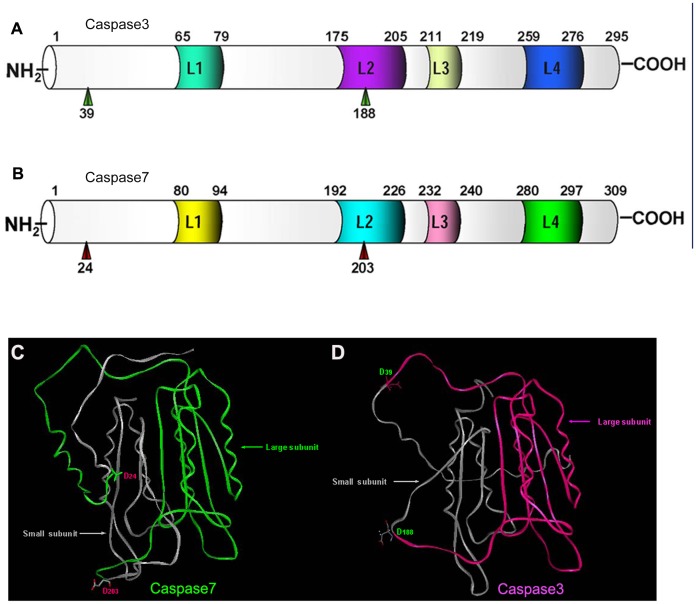
Predicted protein structures of Caspase3 and Caspase7 in *Cynops orientalis*. (A)(B) Schematic representation of the predicted subdomain structure of Caspase3 (A) and Caspase7 (B) in *Cynops orientalis*. The subdomains are shown by the horizontal bar, with the numbers corresponding to the amino acid residues. The cleavage active Aspadine are marked with green or red arrow head, which separate the Caspase3 or Caspase7 into large subunits and small subunits. The four surface loops (L1-L4) that shape the catalytic groove are also labeled with different colors. (C)(D) Cartoon representation of the 3-D structures of Caspase3 and Caspase7. The red color and green stands for the large subunit of Caspase3 and Caspase7, and the gray color indicates the small subunits of Caspase7 and Caspase3. Two cleavage active Asp residues are shown with the form of stick and ball in the cartoon. The 3-D structure prediction was performed by I-TASSER server.

### Caspase Activity Assay

Caspase activities in testes of newts were analyzed using caspase activity assay kits, which were based on the capabilities of caspase-3, -8, -9 to cleave acetyl-Asp-Glu-Val-Asp p-nitroanilide (Ac-DEVD-pNA), acetyl-Ile-Glu-Thr-Asp p-nitroanilide (Ac-IETD-pNA) and acetyl-Leu-Glu -His-Asp p-nitroanilide (Ac-LEHD-pNA) respectively producing yellow formazan product p-nitroaniline (pNA). After heat shock (40°C,2 h), cold exposure (4°C, 12 h), cadmium exposure (36 h) and starvation (one month), newts testes were dissected and rinsed with cold PBS, and then homogenized in ice bath using lysis buffer, with 5 min further extraction in ice bath. The tissue lysates were centrifuged at 18,000×g for 10 min at 4°C. Caspase-3, -8, -9 activities in the supernatant was assayed using the kit (Beyotime, China), and the absorbance at 405 nm was determined to quantify the caspase activities. All the experiments were performed in triplicates, and the caspase activities in the testes of stress-treated newts were presented as percentage of enzyme activity to that in control newts.

**Figure 7:**
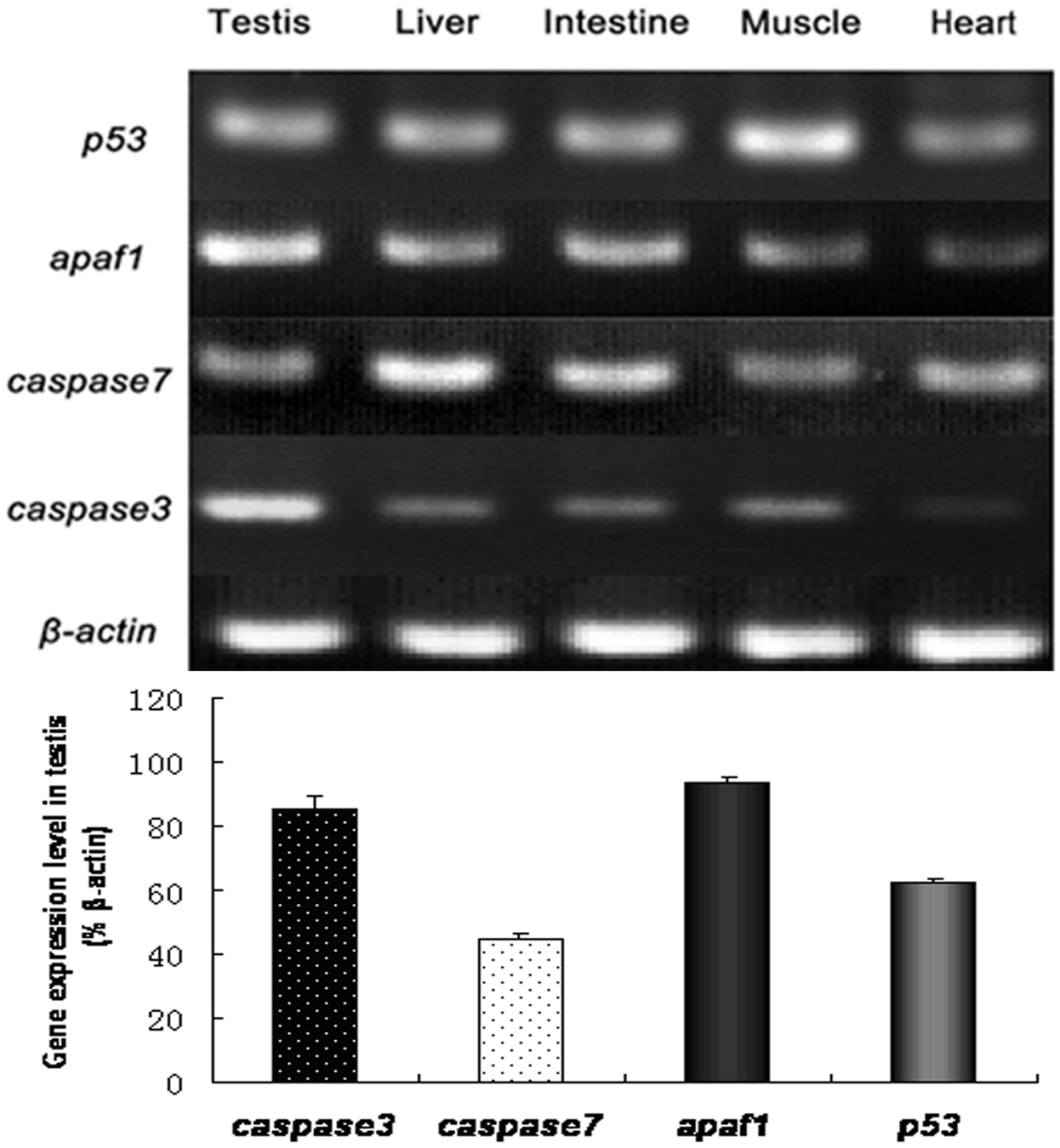
*p53, apaf1, caspase3* and *caspase7* gene expression in various tissues of *Cynops orientalis*. Total RNA was extracted from fresh testis, muscle, liver, intestine and heart, and then transcribed into cDNA for RT-PCR analysis. *β-actin* served as an internal control. *p53, apaf1, caspase3* and *caspase7* are expressed in all the examined tissues. In the newt testis, *Apaf1* and *Caspase3* were expressed at higher level than *p53* and *Caspase7*. The intensity of the signal was analyzed by Tanon-Gis system and the data were processed with SPSS 9.0. The gene expression level in testis were presented as their percentage of *β-actin*. Significance of difference was assessed by SPSS9.0. P<0.05. n  = 3.

### Transmission Electronic Microscopy (TEM)

The newt testis was initially fixed with 2.5% glutaraldehyde and then post-fixed in 1% osmium tetroxide solution at room temperature. After pre-stained with uranyl acetate, the sample was dehydrated with acetone and ethanol before their bedding in Spurr’s resin. The sections were prepared with a Leica Ultracut UC6 Ultramicrotome (Leica Microsystems AG, Wetzlar, Germany). After staining with uranyl acetate and lead citrate, the sections were observed under transmission electronic microscopy (PHILIPS TECNAI 10).

### Statistical Analysis

All data obtained in real-time RT-PCR and caspase activity assay were analyzed using EXCEL and SPSS 9.0 software and presented as mean±standard error. Student’s T-test was used to evaluate the significant difference of gene expression levels and caspase activities between experimental group and control group. It was considered to be statistically significant when P value <0.05.

**Figure 8:**
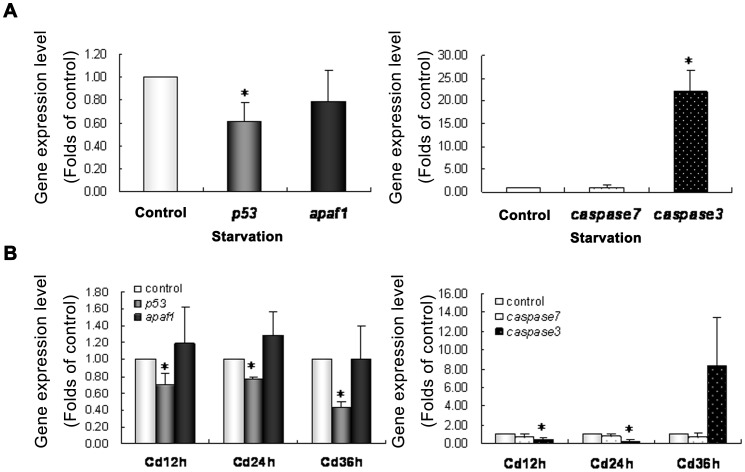
*p53, Apaf1, Caspase3* and *Caspase7* gene expression in the testis of *Cynops orientalis* after starvation and cadmium exposure. (A)(B) gene expression level after starvation (one month).(C)(D) gene expression level after cadmium exposure determined by real-time reverse transcription-PCR. Messenger RNA levels in experimental group were presented as the folds of gene expression level in control group, which was set as 1.0. All data were expressed as Means±SD, and T-test in SPSS9.0 applied to analyze the significance of differences between experimental group and control group. * P<0.05 when compared with control. n  = 3.

## Results

### The cDNA Sequences of p53, Apaf1, Caspase3 and Caspase7

We cloned the full-length cDNA of *p53*, *Apaf1*, *Caspase3* and *Caspase7*. *p53* (HM627863) has a full length of 1588 bp, including a 1179 bp open reading frame (ORF) encoding a putative protein of 392 amino acids (Aa) with the molecular weight of 44 KDa. The *Apaf1* (JQ320086) has a full length of 4313 bp, including a 3747 bp ORF encoding a putative protein of 1248Aa with a molecular weight of 140 KDa. The *Caspase3* (JQ320088) has a full length of 1218 bp with an 888 bp ORF encoding a putative protein of 295Aa with a molecular weight of 33 KDa. The *Caspase7* (JQ320087) has a full length of 2280 bp with an 897 bp ORF encoding a putative protein of 269Aa with a molecular weight of 34 KDa.

**Figure 9:**
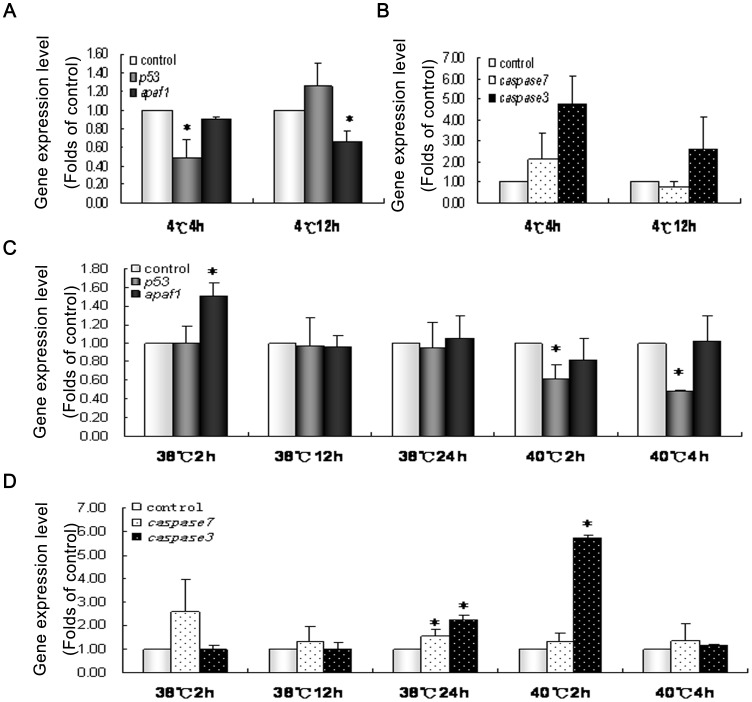
*p53, Apaf1, Caspase3* and *Caspase7* gene expression after stress treatment. The gene express pattern in the testis of Cynops orientalis after cold exposure at (4°C 4 h and 12 h) and heat exposure (38°C 2 h, 12 h and 24 h; 40°C 2 h and 4 h) determined by real-time reverse transcription-PCR. Messenger RNA levels in experimental group were presented as the folds of gene expression level in control group, which was set as 1.0. All data were expressed as Means±SD, and T-test in SPSS9.0 applied to analyze the significance of differences between experimental group and control group. * P<0.05 when compared with control. n  = 3.

### Protein Characterization of p53, Apaf1, Caspase3 and Caspase7

#### Apaf1 and p53

Multiple protein alignment of Apaf1 in *Cynops orientalis* with its homolog demonstrated that it owned 61.4% identity with its homolog in *Homo sapiens*, 55.7% in *Xenopus laevis*, 51.2% in *Danio rerio*, and 59.9% in *Mus musculus* (Figure 1). Protein structure prediction of Apaf1 showed Apaf1 consisted of three functional subdomains: the N-terminal caspase recruitment domain (CARD) (1–89aa), the nucleotide-binding and oligomerization domain (NOD) (90–426) and the C-terminal WD-40 domain. These functional subdomains of Apaf1 were indicated with arrows in its predicted 3-D structure (Figure 2).

Multiple protein alignment of p53 in *Cynops orientalis* with its homolog showed that it had 53.4% identity with its homolog in *Danio rerio*, 49.6% in *Homo sapiens*, 48.5% in *Mus musculus* and 52.8% in *Xenopus laevis*. Several amino acids (Ser235, Arg242, Arg267, Ala270, Cys271 and Arg274) that were essential for DNA binding are found conserved in this protein (Figure 3). The subdomains prediction showed p53 protein owned four functional domains: N-terminal transactivation domain (1–28Aa), proline-rich domain (58–101Aa), DNA binding domain (102–293Aa), tetramerization domain (315–357Aa) and C-terminal domain, which were indicated with arrows in predicted p53 3-D structure (Figure 4).

**Figure 10:**
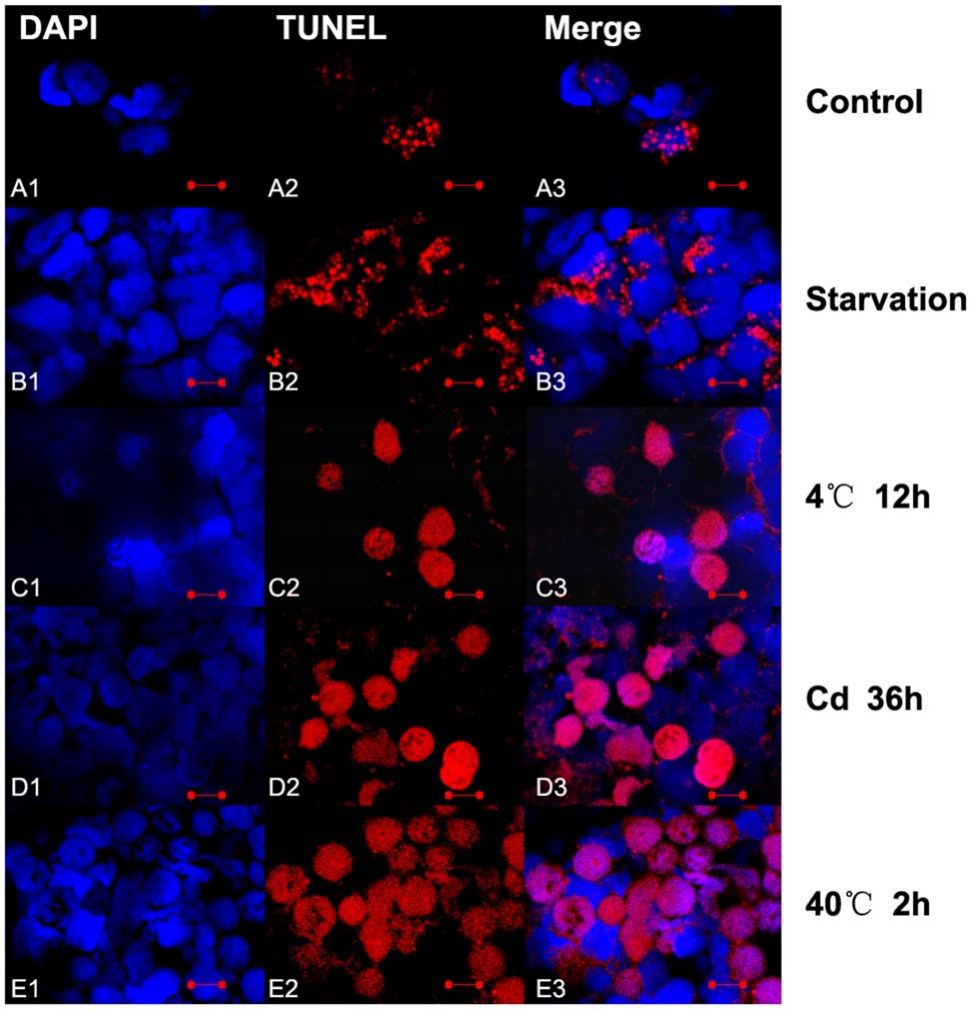
Immunofluorescence analysis of spontaneous apoptosis and stress induced apoptosis in newt early spermatids. The apoptotic germ cells were determined by TUNEL kit (red signal) (Beyotime, China) and the nucleus were dyed with DAPI (Blue signal). Apoptotic sperms were found in normal newt testis (A1-3), and severer mature sperm apoptosis detected under various stress including starvation (B1-3), cold exposure(4°C for 12 h) (C1-3), cadmium exposure (5 mg/Kg body weight for 36 h) (D1-3) and heat exposure (40°C for 2 h) (E1-3). The scale bar is 10 µm.

**Figure 11:**
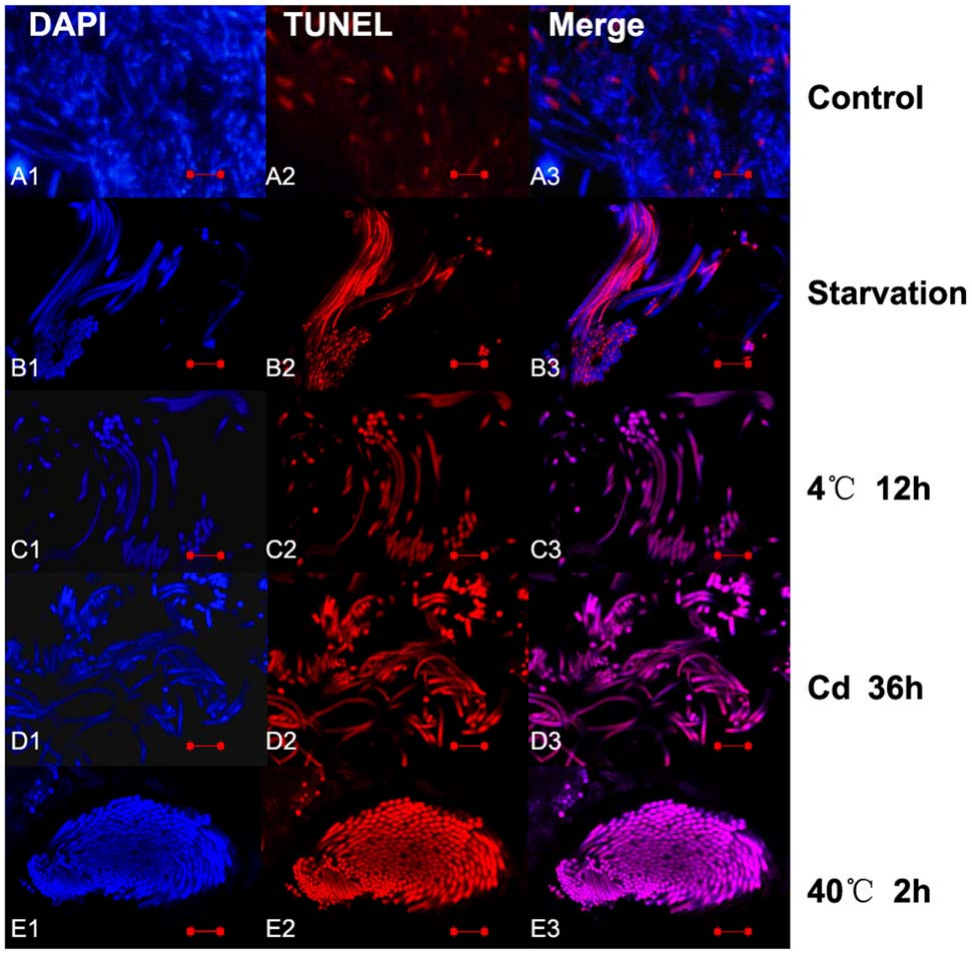
Immunofluorescence analysis of spontaneous apoptosis and stress induced apoptosis in newt mature sperms. The apoptotic germ cells were determined by TUNEL kit (Beyotime, China) and the nucleus were dyed with DAPI. Apoptotic sperms were found in normal newt testis (A1-3), and severer mature sperm apoptosis detected under various stress including starvation (B1-3), cold exposure(4°C for 12 h) (C1-3), cadmium exposure (5 mg/Kg body weight for 36 h) (D1-3) and heat exposure (40°C for 2 h) (E1-3). The scale bar is 10 µm.

**Figure 12:**
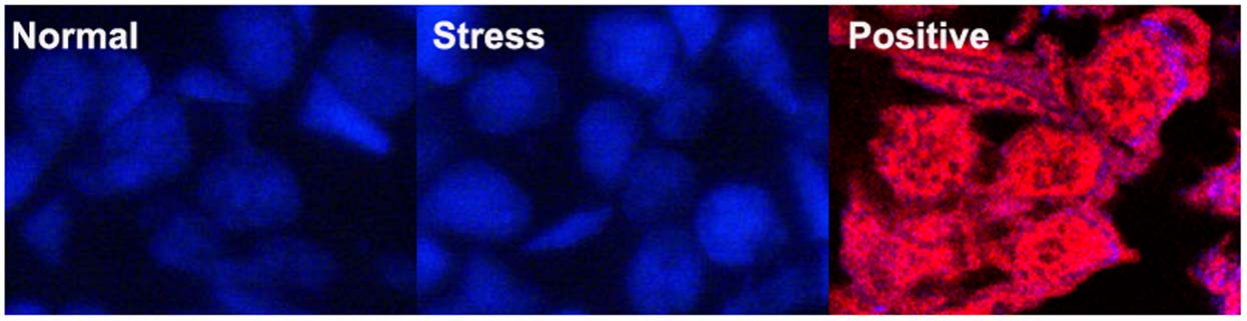
Immunofluorescence analysis on apoptosis. No apoptosis occurred to spermatogonia in normal newt testis and stress treated newt. The apoptotic germ cells were determined by TUNEL kit (red signal) (Beyotime, China) and the nucleus were dyed with DAPI (Blue signal). No apoptotic spermatogonia were found in normal newt testis (A) and stress treated newts (B) while severe apoptotic signal was in spermatogonia in the testis treated with DNase I in the TUNEL positive preparation Kit (Beyotime, China). The scale bar is 10 µm.

#### Caspase3 and caspase7

Multiple protein alignment of Caspase3 and Caspase7 in *Cynops orientalis* with their homolog showed that Caspase3 in *Cynops orientalis* have 56.2% identity with *Danio rerio* homolog, 54.4% with *Xenopus laevis*, 57.6% with *Homo sapiens* and 56.3% with *Mus musculus.* Caspase7 had 67.6% identity with *Danio rerio* homolog, 64.6% with *Xenopus laevis*, 62.6% with *Homo sapiens* and 62.3% with *Mus musculus.* His134 and Cys176 in Caspase3, and His149 and Cys191 in Caspase7 were predicted to be critical for catalytic activity and they were conserved compared with their homolog (Figure 5). The putative cleavage sites at Asp39 and Asp188 in Caspase3, and Asp24 and Asp30 in Caspase7 were also highly conserved, at which Caspase3 and Caspase7 can be separated into functional large subunit and small subunit respectively (Figure 5). The predicted subdomain structures of putative Caspase3 and Caspase7 in *Cynops orientalis* were very similar. They shared 50.6% identity, and both of them had conserved Asp cleavage sites and four loops that contributed to catalytic groove conformation. Additionally, the predicted 3-D structures of Caspase3 and Caspase7 also had high similarity (Figure 6).

### Tissue specific Gene Expression of p53, Apaf1, Caspase3 and Caspase7

RT-PCR result showed that *p53*, *Apaf1*, *Caspase3* and *Caspase7* were expressed in various examined tissues. In the testis, *Caspase3* and *Apaf1* were expressed at slightly higher level than *p53* and *Caspase7* (Figure 7).

**Figure 13:**
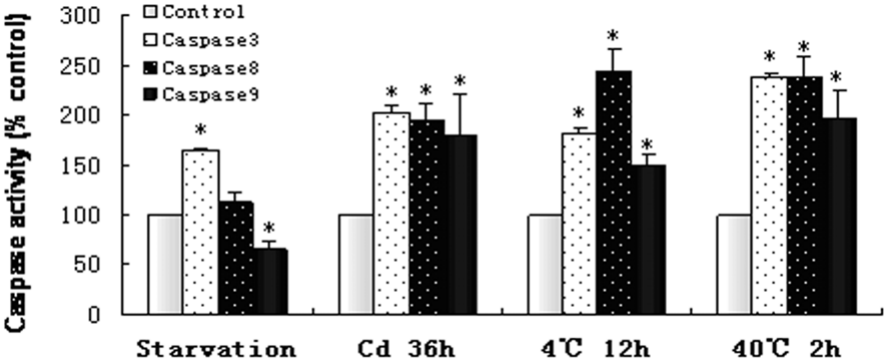
Caspase-3, -8, -9 activities in the testis of newts treated with various stress. The Caspase activities in the testis of newts were analyzed after stress treatment including starvation (one month), cadmium injection (36 h), cold exposure (4°C, 12 h) and heat exposure (40°C, 2 h). Caspase-3, -8, -9 activity was measured by colorimetric assays based on caspase-3, -8, -9 to change Ac-DEVD-pNA, Ac-IETD-pNA and Ac-LEHD-pNA into a yellow formazan product (pNA), respectively. The caspase activities in stress exposure groups were presented as the percentage of enzyme activity in the control group. Value represents the Mean±SD, and T-test in SPSS9.0 applied to analyze the significance of differences between stress treated group and control group. * P<0.05 when compared with control. n  = 3.

**Figure 14:**
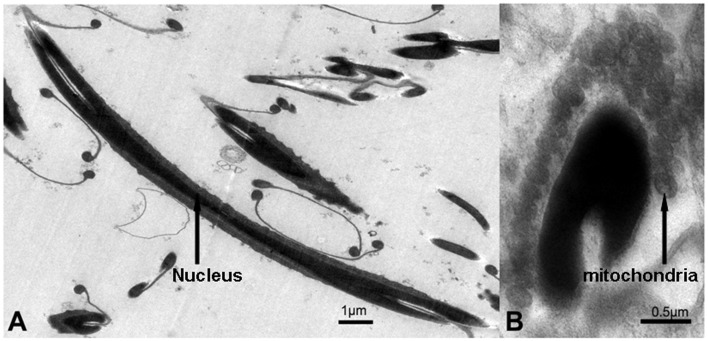
Ultrastructure of newt sperm. (A) Longitudinal section of the mature sperm. The nucleus is exceedingly long and thin (white arrow). Scale bar:1 µm (B) Cross section of the mature sperm nucleus. A large number of mitochondria were arranged around the nucleus (white arrow). Scale bar: 0.5 µm.

### Gene Expression of p53, Apaf1, Caspase3 and Caspase7 in the Testis after Stress Treatment

In the testes of starved newts, gene expression of *p53* was slightly but significantly decreased while *Apaf1* didn’t showed significant alteration. Notably *Caspase3* showed a more than 20-fold significant increase in its gene expression whereas *Caspase7* show no significant change (Figure 8 A and B). After cadmium exposure for 12 h, 24 h and 36 h, the expression of *p53* showed slight but significant decrease while *Apaf1* was slightly increased though without significance. Gene expression of *Caspase3* and *Caspase7* were decreased after cadmium exposure for 12 h and 24 h, but only the change of *Caspase3* mRNA level was significant. After 36 h exposure to cadmium, *Caspase3* gene expression was increased by about 8 fold though without significance, whereas no significant alteration of *Caspase7* was detected (Figure 8C and D).

After 4 h exposure to cold, gene expression of *p53* in newt testis was significantly decreased whereas its expression was elevated after 12 h though without significance. Differently the gene expression of *Apaf1* were decreased but only showed significant difference after cold exposure for 12 h. Gene expression level of *Caspase3* was greatly increased after cold exposure though without statistical significance whereas *Caspase7* didn’t showed significant change after cold exposure (Figure 9A and B). In heat shock group, expression of *p53* showed significant decrease after exposure to 40°C, and no significant change was found after exposure to 38°C. *Apaf1* was only significantly increased after exposure to 38°C for 2 h, without any significant changes in other groups. *Caspase3* were significantly increased after heat shock at 40°C for 2 h and 38°C for 24 h without no significant alterations in other groups. *Caspase7* was only significantly increased in the testes of newts exposed to 38°C for 24 h (Figure 9C and D).

### TUNEL Analysis of Germ Cell Apoptosis in Newt Testis

TUNEL assay showed spontaneous germ cell apoptosis occurred in the testis of normal newts, and apoptotic early spermatids (Figure 10A1-3) and mature sperms (Figure 11A1-3) were detected. Compared with normal newts, TUNEL results showed severer germ cell apoptosis occurred to early spermatids and mature sperm in the testis of newts treated by starvation (Figure 10B1-3, Figure 11B1-3), cold exposure (4°C for 12 h) (Figure 10 C1-3, Figure 11 C1-3), cadmium exposure (36 h) (Figure 10 D1-3, Figure 11 D1-3) and heat shock (40°C for 2 h) (Figure 10 E1-3, Figure 11 E1-3 ). But no apoptotic spermatogonia were observed in the testes of normal newts and stress-treated newts (Figure 12 A and B). In positive TUNEL control sections, strong spermatogonia apoptosis was detected (Figure 12 C).

**Figure 15:**
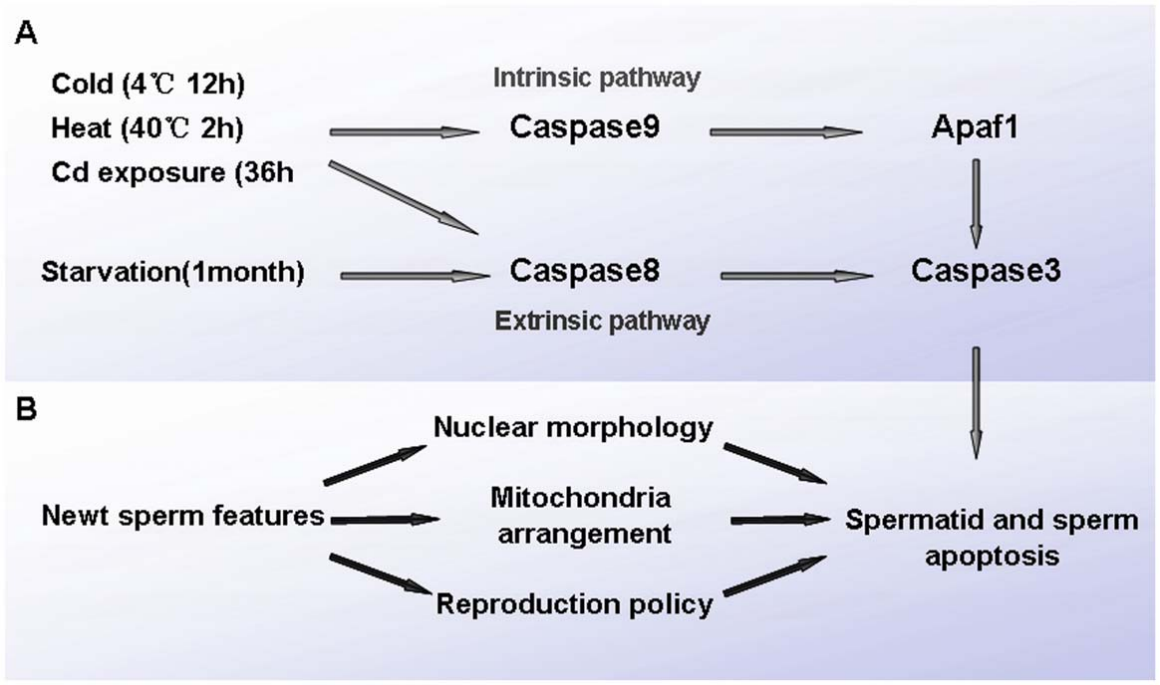
Schematic representation of possible mechanism for the stress triggered germ cell apoptosis in Chinese fire-belly newt, *Cynops orientalis*. (A) Potential pathways initiated by various stress in germ cell apoptosis. Cold exposure, cadmium exposure and heat exposure probably induced germ cell apoptosis via both extrinsic pathway and intrinsic pathway, but starvation likely only via extrinsic pathway. (B) Hypothesized sperm features and unique reproduction policy responsible for high occurrence of spermatid and sperm apoptosis. The nuclear morphology and mitochondria arrangement may be the physiological basis for observed high frequency of spermatid and sperm apoptosis, and a unique reproduction policy of newts may also account for this phenomenon.

### Caspase Activity Alteration in Newt Testes after Stress Treatment

In response to starvation, Caspase3 and Caspase8 activities in newt testes were increased to 166% and 112% while Caspase9 activity was decreased to 65% of the control. Under cadmium exposure, Caspase-3, -8, -9 activities were significantly elevated to 202%, 194% and 180% of the control. The activities of Caspase3, Caspase8 and Caspase9 were significantly increased to182%, 245% and 149% after cold exposure (4°C 12 h), and elevated to 238%, 239% and 196% after heat shock (40°C 2 h) (Figure 13).

### Ultrastructure of Newt Mature Sperm

The TEM results showed that the nucleus of newt mature sperm was long and narrow shaped (Figure 14 A). A large number of mitochondria were observed arranged surrounding the sperm nucleus (Figure 14 B).

## Discussion

### p53, Apaf1, Caspase3 and Caspase7 were Conserved in Chinese Fire-belly Newt, *Cynops Orientalis*

We have cloned the full-length cDNA of *p53*, *Apaf1*, *Caspase3* and *Caspase7* from the testis of Chinese fire-belly newt, *Cynops orientalis*. p53 and Apaf1 protein share about 50% identity with their homolog, and they have conserved functional subdomains. The DNA binding domain of p53 is highly conserved and six key amino acids essential for binding capability are totally the same among the examined homolog [58]. Besides, the transactivation domain and profine-rich domain conferred p53 possible capability in gene expression regulation while tetramerization domain indicated p53 probably became functional in a tetramer form [58], [59]. As for Apaf1, CARD is an essential functional domain because it is responsible for recruiting procaspase9 for apoptosome formation [24], [25], [60]. The NOD domain is involved in Apaf1 oligmerization into apoptosome [61], and the C-terminal 12 WD40 repeats contributed to cytC binding to initiate downstream cascade of caspases [24], [25], [61], [62], [63]. The 3-D structure of p53 and Apaf1 was quite similar to their counterparts in mammals [58], [64]. Base on the highly conserved subdomain structures and similar 3-D structures, p53 and Apaf1 in newts probably performed similar functions to their homolog in mammals.

Caspase3 and Caspase7 showed 54%–57% and 62%–67% identity with their homolog respectively. As critical cleavage sites, Asp24/Asp203 (Caspase7) and Asp39/Asp188 (Caspase3) are conserved, revealing a similar cleavage mechanism. Besides, being key catalytic residues, the highly conserved His134/Cys176 (Caspase3) and His149/Cys191 (Caspase7) indicated an similar catalytic performance [65], [66]. As effector caspases, Caspase3 and Caspase7 in *Cynops orientalis* have highly similar subdomain structure and 3D structures, which further demonstrated that their similar performance in cell apoptosis resulted from resembled structures [67].

RT-PCR results showed that *p53*, *Apaf1*, *Caspase3* and *Caspase7* were expressed in various examined tissues. In the testis, *Caspase3* and *Apaf1* were expressed at higher level than *p53* and *Caspase7*, which indicated more active *Caspase3* and *Apaf1* in newt testis. *Caspase3* and *Caspase7* have been found expressed during normal spermatogenesis, and play important functions in spontaneous germ cell apoptosis [23]. *p53* and *Apaf1* were also proved expressed in testis, and they are critical for sperm development and male fertility [23], [68], [69]. Knockout or overexpression of *p53* would result in abnormal spermatogenesis [22], [23] while knockout of Apaf1 could induce male infertility [70].

### Spontaneous Germ Cell Apoptosis Occurred in Newt Testis

During normal spermatogenesis, spontaneous germ cell apoptosis commonly occurred to spermatogonia and spermatocytes in many mammalian species including rats, hamsters, mice and humans [2], [3], [4], [5], [6], [7], [8], [9], [45], [71], [72,]. Since the spermatogonia and spermatocytes were active in DNA replication, which made them vulnerable to damage [21], [45], [73], [74], [75]. In some cases, apoptosis even occurred to spermatids or sperm, though the frequency is low [2], [76]. In the present study, we only observed apoptotic spermatids and sperm without apoptotic spermatogonia and spermatocytes detected, whereas severe apoptotic spermatogonia were observed in positive control sections. The result indicated the intriguing germ cell apoptosis in newts was probably resulted from specificity of this species. The spontaneous germ cell apoptosis in newt testis may be responsible for clearing off abnormal spermatogenic cells and ensuring production of high quality sperm [27], [28], [30], [77].

### Caspase3 and Caspase7 are Involved in Stress-induced Germ Cell Apoptosis

When spermatogenesis was threatened by environmental or physiological stress, stress-induced germ cell apoptosis was often initiated to remove abnormal germ cells [78], [79], [80]. For instance, hormone deprivation, heat shock, low temperature, starvation and heavy mental exposure could aggravate germ cell apoptosis in testes [50], [77], [81], [82]. In our research, negative effects were exerted by cadmium exposure (36 h), heat shock (40°C 2h), cold exposure (4°C 12h) and starvation (one month) on germ cell maturation in newt, leading to severer spermatid and sperm apoptosis. Obviously, the germ cell development in *Cynops orientalis* was vulnerable to stress, and germ cell apoptosis had been triggered by temperature stress, starvation and heavy metal exposure.

Caspase3, Caspase6 and Caspase7 were three important effector caspases that directly determined cell death in cell apoptosis signaling pathway [66], [83]. Previous researches on newts have demonstrated that high caspase activity in toxicant-treated testes was responsible for chromatin condensation and apoptotic body formation [49]. In our study, after stress treatment, not only gene expressions of *Caspase3* or *Caspase7* in newt testis were obviously elevated, but also the Caspase3 enzyme activity in the testis was significantly increased. Therefore, the stress-induced germ cell apoptosis was dependent on both gene expression and enzyme activation of Caspase3.

### Possible Mechanism for Starvation-induced Germ Cell Apoptosis

Nutrients are essential for male reproduction performance, for it can supply energy like glucose and growth factors that tightly correlates with cell metabolism, growth and proliferation [84], [85]. Without sufficient growth factor and glucose, cells firstly reduced its metabolism and ceased proliferation, and further nutrients deprivation would initiate cell apoptosis [86], [87], [88], [89], [90], [91], [92], [93]. In our study, after one-month food deprivation, we found severer germ cell apoptosis occurred in the testis of *Cynops orientalis* compared with control newt, which probably resulted from serious shortage of growth factor and glucose. It was demonstrated that cell apoptosis triggered by nutrients deprivation was caspase-dependent, and executed mainly by intrinsic mitochondria-dependent pathway [85], [94], [95], [96]. In detail, nutrients deprivation could change cellular metabolism, and disrupted the overall balance between proapoptotic and antiapoptotic Bcl-2 family members. Then mitochondrial physiology would be altered to facilitate cytC release into the cytosol. Together with Apaf1 and procaspase9, cytC was assembled into the apoptosome to activate caspase9 that further activated effector caspases to induce cell death [93], [97], [98]. In addition, p53 was elucidated to be involved in starvation-induced cell apoptosis [99], and Fas-dependent extrinsic pathway proved critical for germ cell apoptosis after growth factor withdrawal [100]. However, in our study, after one month starvation, either the gene expression of *Apaf1* and *p53* or Caspase9 enzyme activity in the testis of *Cynops orientalis* was significantly decreased. Therefore intrinsic pathway was probably not involved in starvation-induced germ cell apoptosis. In contrast, Caspase8 enzyme activity in the testis was slightly elevated after starvation treatment, which indicated that the extrinsic pathway might participate in the starvation-induced germ cell apoptosis in *Cynops orientalis*.

### Possible Mechanism for Cadmium-induced Germ Cell Apoptosis

Cadmium is one of the most toxicants in terrestrial and aquatic environment, and both cadmium pollution and cadmium injection into testis can induce testicular impairment, germ cell death, poor semen quality and even male infertility [80], [101], [102], [103], [104], [105], [106], [107]. Cadmium triggered germ cell apoptosis by disrupting endocrine secretion and enhancing reactive oxygen species (ROS) production. On the one hand, cadmium is an endocrine disruptor that can affect functions of Leydig cell and inhibit testicular testosterone secretion [4], [35], [108], [109], [110], [111], [112], [113], both of which were essential for spermatogenesis [2], [114], [115]. Once the testosterone was inhibited by cadmium, massive testicular germ cell apoptosis would occur [101], [106]. Fas and FasL have been elucidated to play pivotal roles in germ cell apoptosis after testosterone deprivation and cadmium exposure, with their gene expression increased both in germ cells and Sertoli cells [32], [35], [36], [77], [80], [103], [116], [117]. In this study, the enzyme activity of Caspase8 in the testis was elevated by 100%, and it demonstrated that the cadmium exposure probably disrupted the hormone secretion and the extrinsic cell death pathway was initiated. On the other hand, cadmium could induce germ cell apoptosis by enhancing generation of ROS like superoxide ion, hydroxyl radicals and hydrogen peroxide, and these oxidative stress could result in peroxidation, mitochondrial dysfunction or DNA damage in germ cell and oxidative damage in Leydig cells [80], [118], [119], [120], [121], [122], [123], [124]. Oxidative stress could induce p53 mediated cell apoptosis in the intrinsic mitochondria-dependent pathway [125], [126], [127], [128]. The Caspase9 enzyme activity in the testis was raised by 80%, and probably the mitochondria-dependent intrinsic pathway was also involved in the cadmium-induced germ cell apoptosis. As the key participant of intrinsic cell death pathway, Apaf1 probably participate in the cell apoptosis in spite of no elevation in its gene expression.

Under oxidative stress, p53 could be activated to promote reduced glutathione generation and sestrins expression, thus protecting DNA from damage by decreasing ROS [129]. But we found that gene expression of *p53* was reduced after cadmium exposure, and previous study also showed cadmium suppressed *p53* gene expression in the testes and liver [101], [130]. So we deduced that ROS induced by cadmium may result in devastating DNA damage, which directly lead to cell apoptosis in a p53-independent pathway.

### Possible Mechanism for Heat- and Cold-induced Germ Cell Apoptosis

Suitable environmental temperature is important for male reproduction. Heat shock can induce germ cell death and even male infertility while cold temperature leads to abnormal sperm production [53]. In adult rat testis, heat shock (43°C for 15 min) caused germinal epithelium damage, abnormal Sertoli cell and germ cell death [11], [131], [132] that mainly occurred to meiotic spermatocytes and early spermatids [11]. The intrinsic mitochondria-dependent pathway proved critical for heat-induced germ cell apoptosis, in which Bcl-2 family members played important functions [77], [81], [133], [134], [135]. Bax induced the cyt C release from the mitochondria to initiate the activation of Caspase9, which eventually activated downstream effector caspases (caspase3, 6 and 7) leading to cell death [81], [136]. Moreover, Fas-dependent extrinsic pathway was also involved in heat-induced testicular germ cell apoptosis in cryptorchid testis [31]. We detected that enzyme activities of Caspase8 and Caspase9 in newt testis were significantly enhanced by 139% and 96% respectively after heat shock, indicating the involvement of extrinsic and intrinsic pathway in the germ cell apoptosis. Though without significant alteration, Apaf1 also should be involved in heat-induced germ cell apoptosis regarding its key status in intrinsic pathway. Though p53-dependent apoptosis was reported to be responsible for the initial phase of germ cell loss in experimental cryptorchid testis [31], *p53* gene expression was significantly decreased in our study, indicating it probably didn’t participate in this process.

Low temperature could trigger germ cell apoptosis mainly by reducing FSH secretion, which was critical for male reproduction especially in seasonally breeding animals [53]. FSH not only supported proliferation and maturation of Sertoli cells [137], but also maintained spermatogonia viability and proliferation [52], [138]. Additionally, FSH could inhibit PRL induced spermatogonia cell death both *in vivo* and *in vitro* [51], [53]. In newts, spermatogonia apoptosis occurred after cold exposure for one week (12° and 8°C) whereas no degeneration was found in newts kept at 22°C. Notably, cold triggered germ cell death was significantly suppressed by FSH at 12°C and 22°C but not at 8°C [50], [53], indicating the irremediable germ cell apoptosis induced by lower temperature. Therefore severe germ cell apoptosis triggered by cold exposure (4°C) in our research to a big extent resulted from significant decrease in FSH secretion. It was elucidated that FSH can determine cell destiny by regulating several important proteins, including Survivin, PDCD6 and DR5, all of which were key members in intrinsic pathway and extrinsic pathway in cell apoptosis. High level of FSH could upregulate the expression of Survivin and suppress the expression of PDCD6 and DR5 [139], thus to promote cancer development and inhibit cell apoptosis. When FSH level in the testis was decreased after cold exposure, the expression of Survivin was probably reduced while the inhibition of PDCD6 and DR5 was terminated. In this way, both extrinsic and intrinsic pathway could participate in cold-induced germ cell apoptosis, which was evidenced by the significantly elevated enzyme activities of Caspase8 (150%) and Caspase9 (50%) in our research. Moreover increased gene expression of Apaf1 indicated it probably participate in cell apoptosis while p53 may not for its decreased gene expression.

### Possible Mechanism for Intriguing Sperm Apoptosis

The most intriguing phenomenon in our study is that germ cell apoptosis commonly occurred to spermatids and mature sperm, but not to spermatogonia and spermatocytes. Differently, other researches on newts only found apoptotic spermatogonia, spermtocytes and only abnormal mature sperm [47], [48], [49]. In previous researches, apoptotic mitotic diploid spermatogonia and meiotic primary spermatocytes were most common, and then was the apoptotic spermatids, either in spontaneous germ cell death or stress-induced cell apoptosis [3], [45], [140], [141], [142], [143]. Only in recent years, more and more researches reported the occurrence of mature sperm apoptosis [76], [144], [145]. Several key apoptotic proteins were found in mature spermatozoa. For instance, in ejaculated mature sperm, functional p53 was localized in 43% of semen samples, and PARP in 75–100% of the sperm [144], [146]. Moreover, active caspase1, caspase3 and caspase8 were found predominantly in the postacrosomal region of live sperm, and active caspase9 was particularly localized in the midpiece [76]. In human sperm, procaspases 8 and 9 and their active forms, together with caspase3, were also observed [146]. Furthermore, sperm fractions with low motility exhibit higher levels of active caspase3 compared with high-motility fractions [147]. Active caspase3 found in the sperm midpiece were closely associated with DNA fragmentation in low motility semen specimen, suggesting that caspase-dependent apoptotic mechanisms could originate in the cytoplasmic droplet or within mitochondria and function in the nucleus [148]. The activation of caspases in low-motility sperm samples may be attributed to the role played by cytC/Apaf-1 complex, which can activate caspase9 that was followed by activation of downstream caspase3, caspase6 and caspase7 [145].

We hypothesized that several physiological features of Chinese fire-belly newts might account for why mature sperm were easily involved in cell apoptosis. Above all, newt mature sperm had a long and narrow shaped nucleus which may be quite vulnerable to environmental stress (Figure 14 A). Moreover, similar to the mitochondria in midpiece of mammalian sperm, a large number of mitochondria were arranged surrounding the nucleus of newt sperm (Figure 14 B), which provided a possible platform for mitochondria-dependent cell death. Finally, it probably resulted from the specificity of this species. Without stringent controlling of early stage spermatogenic cell proliferation, the newts can firstly ensure high production of spermatids and sperm, and at the late stage of spermatogenesis, the quality of spermatids and sperm could be strictly controlled by removing abnormal spermatids and sperm via germ cell apoptosis.

### Conclusion

From the results we can get several conclusions 1) p53, Apaf1, Caspase3 and Caspase7 are conserved in the testis of *Cynops orientalis*, and Caspase3, Caspase7 and Apaf1 were probably involved in the stress-triggered germ cell death while the roles played by p53 needs further investigation; 2) Spontaneous germ cell apoptosis occurred during spermatogenesis; 3) cadmium exposure, cold exposure and heat shock triggered severer apoptosis of spermatids and sperm probably both via extrinsic pathway and intrinsic pathway while starvation induced germ cell apoptosis possibly mainly via the intrinsic pathway (Figure 15).
